# Exercise training in childhood-onset systemic lupus erythematosus: a controlled randomized trial

**DOI:** 10.1186/ar4205

**Published:** 2013-03-26

**Authors:** Danilo ML Prado, Fabiana B Benatti, Ana L de Sá-Pinto, Ana P Hayashi, Bruno Gualano, Rosa MR Pereira, Adriana ME Sallum, Eloisa Bonfá, Clovis A Silva, Hamilton Roschel

**Affiliations:** 1 Division of Rheumatology, School of Medicine, University of Sao Paulo, Av. Dr. Eneas de Carvalho Aguiar, 255 - Sao Paulo, SP, CEP 05403-000, Brazil; 2 School of Physical Education and Sport, University of Sao Paulo, Rua Professor Mello Moraes, 65- Sao Paulo, SP, CEP 05025-010, Brazil; 3 Pediatric Rheumatology Unit, Children's Institute, School of Medicine, University of Sao Paulo, Av. Dr. Arnaldo, 455 - Cerqueira César, Sao Paulo, SP, CEP 01246-903, Brazil

## Abstract

**Introduction:**

Exercise training has emerged as a promising therapeutic strategy to counteract physical dysfunction in adult systemic lupus erythematosus. However, no longitudinal studies have evaluated the effects of an exercise training program in childhood-onset systemic lupus erythematosus (C-SLE) patients. The objective was to evaluate the safety and the efficacy of a supervised aerobic training program in improving the cardiorespiratory capacity in C-SLE patients.

**Methods:**

Nineteen physically inactive C-SLE patients were randomly assigned into two groups: trained (TR, *n *= 10, supervised moderate-intensity aerobic exercise program) and non-trained (NT, *n *= 9). Gender-, body mass index (BMI)- and age-matched healthy children were recruited as controls (C, *n *= 10) for baseline (PRE) measurements only. C-SLE patients were assessed at PRE and after 12 weeks of training (POST). Main measurements included exercise tolerance and cardiorespiratory measurements in response to a maximal exercise (that is, peak VO_2_, chronotropic reserve (CR), and the heart rate recovery (ΔHRR) (that is, the difference between HR at peak exercise and at both the first (ΔHRR1) and second (ΔHRR2) minutes of recovery after exercise).

**Results:**

The C-SLE NT patients did not present changes in any of the cardiorespiratory parameters at POST (*P *> 0.05). In contrast, the exercise training program was effective in promoting significant increases in time-to-exhaustion (*P *= 0.01; *ES *= 1.07), peak speed (*P *= 0.01; *ES *= 1.08), peak VO_2 _(*P *= 0.04; *ES *= 0.86), CR (*P *= 0.06; *ES *= 0.83), and in ΔHRR1 and ΔHRR2 (*P *= 0.003; *ES *= 1.29 and *P *= 0.0008; *ES *= 1.36, respectively) in the C-SLE TR when compared with the NT group. Moreover, cardiorespiratory parameters were comparable between C-SLE TR patients and C subjects after the exercise training intervention, as evidenced by the ANOVA analysis (*P *> 0.05, TR vs. C). SLEDAI-2K scores remained stable throughout the study.

**Conclusion:**

A 3-month aerobic exercise training was safe and capable of ameliorating the cardiorespiratory capacity and the autonomic function in C-SLE patients.

**Trial registration:**

NCT01515163.

## Introduction

Systemic lupus erythematosus (SLE) is a life-long systemic autoimmune disease with a large variability in its clinical course. The onset of SLE during childhood and adolescence represents 10 to 20% of all SLE cases [1] and is associated with a more severe disease course compared to adult SLE. Due to earlier diagnosis as well as significant advances in the management of childhood-onset SLE (C-SLE), mortality rates have greatly decreased, with an estimated 5- to 10-year survival rate of over 85% [2]. As a consequence, there has been a great increase in long-term co-morbidities, particularly cardiovascular disease (CVD), which is currently considered a major cause of long-term mortality in C-SLE patients [3].

Both disease-related (namely, long-term corticosteroid use, systemic inflammation, and autonomic dysfunction) and traditional factors (namely, lipid abnormalities, diabetes mellitus, and hypertension) have been implicated in the increased CVD risk in SLE [4-7]. Additionally, SLE patients commonly present a reduced exercise capacity and physical function [8-12], which may further increase CVD and mortality risk [13].

In this context, exercise training has emerged as a potential non-pharmacological therapeutic strategy to counteract physical dysfunction in adult SLE [8,9,14-16], although no studies have been conducted with C-SLE patients. It is well-known that exercise-induced adaptations largely differ between adults and children. This holds true for a variety of training variables, including (but not limited to) maximal aerobic capacity, mechanical efficiency and economy of movement, anaerobic capacity, exercise recovery, cardiovascular response, strength, immune response, morphological adaptations, detraining, metabolic responses, thermal regulation, etcetera. Such discrepant responses rely on physiological, metabolic, maturational, social, affective, and perceptual characteristics that are unique to pediatric populations [17]. Additionally, it has been also recognized that C-SLE patients may show differential features compared with adult counterparts, especially in regard to clinical symptoms and treatment response [18-21]. In light of these factors, one may suggest that the exercise training-induced outcomes found in the adult SLE population cannot be promptly generalized to C-SLE, thus warranting studies in this specific population.

A few studies have demonstrated the beneficial role of exercise in other pediatric rheumatic diseases, including juvenile dermatomyositis (JDM) [22,23] and fibromyalgia [24]. Furthermore, we recently demonstrated that a three-month supervised aerobic training program was effective in improving the aerobic capacity and the physical function of a 15-year-old boy with C-SLE associated with secondary antiphospholipid syndrome [25]. However, to the best of our knowledge, no longitudinal studies have evaluated the potential therapeutic effects of an exercise training program in the C-SLE population [26].

Thus, the aim of the present study was to evaluate the safety and the efficacy of a 12-week supervised aerobic training program in improving the cardiorespiratory capacity in C-SLE patients.

## Materials and methods

### Experimental design and patients

A 12-week randomized trial was conducted between May 2010 and April 2011 at the Laboratory of Physical Conditioning in Rheumatology of the School of Medicine, University of Sao Paulo, Brazil. This trial was registered at clinicaltrials.gov as NCT01515163. The sample consisted of children and adolescents with C-SLE from the outpatient ambulatory of the Pediatric Rheumatology Division of the Children's Institute of the School of Medicine, University of Sao Paulo, Brazil, who were randomly assigned to participate in a three-month supervised exercise training program (trained, TR) or to remain physically inactive (non-trained, NT). Randomization was accomplished through a block randomization procedure with block sizes of four individuals. Body mass index-, gender- and age-matched healthy children from the surrounding community were recruited as controls (C). The patients fulfilled the revised American College of Rheumatology criteria [27] for C-SLE. The inclusion criteria were: age between 7 and 15 years, and Systemic Lupus Erythematosus Disease Activity Index (SLEDAI)-2K score ≤ 16. Exclusion criteria included neuropsychiatry involvement, hematologic abnormalities, cardiovascular rhythm and conduction disorders, musculoskeletal disturbances that precluded participation in physical exercise, pleuritis, pericarditis, endocarditis, acute kidney failure, use of tobacco, treatment with lipid-lowering drugs, secondary fibromyalgia. Healthy control subjects were not taking any medication. C-SLE patients had not engaged in regular physical activity programs at least 6 months prior to the study. Importantly, the healthy control subjects were engaged only in the physical education classes in school (a 45-minute session twice a week).

At baseline (PRE) and 12 weeks after the intervention (POST), clinical (that is SLEDAI-2K [28] and Systemic Lupus International Collaborating Clinics/ACR Damage Index (SLICC/ACR-DI) [29], and laboratory parameters (namely, erythrocyte sedimentation rate (ESR), C-reactive protein (CRP), complements 3 and 4 (C3 and C4) and anti-dsDNA antibodies), cardiorespiratory measurements in response to a maximal graded exercise, body composition, and bone mineral density (BMD) were assessed in the C-SLE patients. Healthy children and adolescents were assessed for the same aforementioned parameters only at baseline. The study was approved by the Committee of Ethics in Research of the General Hospital of the School of Medicine, University of Sao Paulo, Brazil (CAPPesq) and all of the subjects' parents signed the informed consent.

### Exercise training program

The exercise program consisted of 12 weeks of twice-weekly supervised moderate-intensity aerobic exercise training. Training sessions comprised a 5-minute warm-up followed by 20 to 50 minutes of treadmill aerobic training (a 10-minute increment in the aerobic training volume was applied every four weeks), and 5 minutes of cool-down on the treadmill at a low speed followed by stretching exercises. All of the training sessions were monitored by at least one fitness professional and a Rheumatologist monitored adverse events on a weekly basis. The exercise program was performed in an intra-hospital gymnasium. Aerobic training intensity was set at the corresponding heart rate between the ventilatory anaerobic threshold (VAT) and 10% below the respiratory compensation point (RCP). All of the patients were able to achieve the set aerobic training intensity.

### Cardiorespiratory exercise test

The cardiorespiratory exercise test was performed on a treadmill (Centurion, model 200, Micromed, Brazil), using a maximal graded exercise protocol [30]. The treadmill speed (2.0, 2.5, 3.0, 3.5, 4.0, 4.0, 4.0, 4.0, 5.0, 5.4, 5.9, 6.3, 6.3 mph), or grade (0.0, 0.0, 0.0, 0.0, 0.0, 2.5, 5.0, 7.5, 4.0, 4.0, 4.0, 4.0. 5.8%) was increased every 1 minute. The recovery period was set at 2 minutes. Oxygen consumption (VO_2_) and carbon dioxide output were obtained through breath-by-breath sampling and expressed as a 30-second average using an indirect calorimetry system (Cortex, model Metalyzer III B, Leipzig, Germany). Heart Rate (HR) was continuously recorded at rest, during exercise, and at recovery, using a 12-lead electrocardiogram (Ergo PC Elite, InC. Micromed, Brasília, DF, Brazil). Peak oxygen consumption (peak VO_2_), VAT and RCP were determined according to previously described criteria [30]. The test was deemed complete when despite verbal encouragement, the child was unable to continue exercising. The following criteria were used to define maximal effort: 1) subjective evidence of exhaustion and either 2) peak HR > 190 beats/minute, or 3) maximal respiratory exchange ratio (RER) > 1.00 [31].

### HR response during exercise and recovery

HR response during exercise was evaluated by the chronotropic reserve (CR) as follows [32]:

$$\left(\left(\text{CR}\right)\;=\;\left(\text{Peak HR }-\text{Resting HR}/)208\;-\left(0.7\;*\text{ Age}\right)\;-\text{ Resting HR}\right]\;\times \;100\right).$$

HR recovery was defined as the difference between HR at peak and at both the first (ΔHRR1) and second (ΔHRR2) minutes after exercise. Absolute change (Δ) was used to calculate the difference between the HR at peak exercise and at the first and second minutes after the exercise test. The relative change in HR (Δ%) was calculated for the intervals between rest to VAT, rest to RCP, and rest to peak exercise.

### BMD and body composition

BMD (g/cm^2^) was determined by dual-energy x-ray absorptiometry (DXA), using a Hologic QDR 4500 Discovery densitometer (Hologic Inc., Bedford, MA, USA). Bone area (BA) (cm^2^) was calculated using the software provided with the densitometer. Bone mass was analyzed in the lumbar spine (lumbar vertebrae L1 to L4), total femur, and whole body. The whole-body analysis was conducted without including the head, as it has been described to increase variability in children [33]. To minimize the confounding effect of skeletal size on DXA measurements, a volumetric, three-dimensional approximation of bone density was calculated (that is, bone mineral apparent density (BMAD) in g/cm^3^). BMAD was assessed by dividing the BMD in a given site (the lumbar spine, total femur, or whole body) by the square root of the corresponding bone area (BA) (BMAD = BMD/√BA) [34]. All of the measurements were performed by the same trained technologist. Precision error for BMD measurements was determined according to the standards in the International Society for Clinical Densitometry protocols [35]. Least significant changes with 95% confidence were 0.033 g/cm^2 ^at the lumbar spine, 0.039 g/cm^2 ^at the total femur, and 0.020 g/cm^2 ^at the whole body. Body composition (that is, lean mass and fat mass) was assessed through the same densitometer, using pediatric software.

### Statistical analysis

The data are presented as means and SDs. Fisher's exact test was used to assess baseline differences between the C-SLE groups in the drug regimen. The disease-related parameters (SLEDAI-2K, SLICC/ACR-DI, disease duration, and current and cumulative dose of drugs) at baseline were compared using the Student's *t-*test. After the normality and homogeneity of the variance were confirmed, the dependent variables were compared using a mixed model for repeated measures, assuming groups and time as fixed factors and subjects as random factors. Single degree of freedom contrasts were used to determine significant differences between groups. Finally, effect sizes (ES) were estimated for the POST assessments using the pooled SDs of the two independent samples at POST [36]. The significance level was set at *P *< 0.05. All of the analyses were performed using SAS 9.2, SAS Institute Inc., Cary, NC, USA.

## Results

### Patients

**Figure 1:**
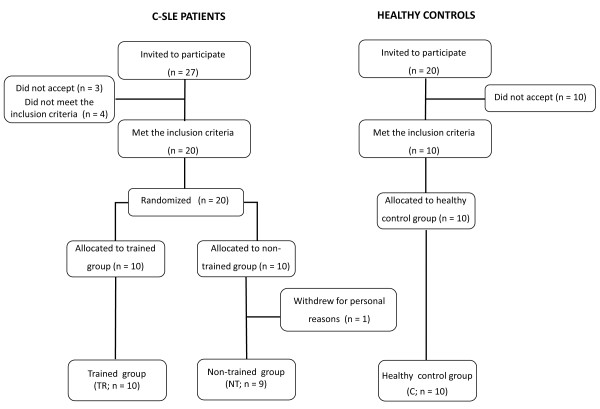
**Flow diagram of the patients**. C-SLE, childhood-onset systemic lupus erythematosus.

Twenty C-SLE patients were randomly assigned to the experimental groups (TR = 10 and NT = 10). One patient from the NT group withdrew from the study due to personal reasons and was excluded from the analysis. Thus, 19 patients were included in the analysis (TR = 10, NT = 9) (Figure 1). Ten healthy subjects were included in the healthy control group (C = 10). Table 1 shows the demographic data of the C-SLE patients and healthy control subjects at baseline. The groups were similar in age, weight, height, BMI values, and drug regime at baseline (*P *> 0.05). The exercise adherence rate was 94.0 ± 7.03%.

**Table 1:** Demographic data at baseline

Variable	C-SLE trained (*n *= 10)	C-SLE non-trained (*n *= 9)	Control (*n *= 10)
Age, years	12.9 (2.3)	13.0 (1.8)	12.0 (1.8)
Body weight, Kg	48.7 (10.1)	49.7 (12.1)	46.6 (14.5)
Height, m	1.48 (0.09)	1.44 (0.11)	1.51 (0.13)
Body mass index, Kg/m^2^	22.3 (3.8)	23.9 (3.4)	21.2 (1.7)
Pubertal stages I/II/III/IV/V, number of individuals	1/2/2/2/3	0/2/3/2/2	3/2/1/2/2
SLEDAI-2K, score	5.3 (5.3)	5.6 (6.4)	NA
SLICC/ACR-DI, score	0.4 (0.7)	0.4 (0.7)	NA
Disease duration, years	3.3 (2.6)	4.3 (2.6)	NA
Current dose of prednisone, mg/d	19.8 (17.0)	15.5 (7.7)	NA
Cumulative dose of prednisone, g	13.7 (6.7)	20.9 (13.0)	NA
Current dose of azathioprine, mg/d	128.6 (39.3)	121.4 (17.2)	NA
Cumulative dose of azathioprine, g	58.2 (32.1)	62.2 (41.1)	NA
Current dose of chloroquine, g/d	205.8 (51.2)	205.6 (51.2)	NA
Cumulative dose of chloroquine, g	129.7 (119.9)	181.3 (121.5)	NA
Drugs, number of patients (%)			
Prednisone	10 (100)	9 (100)	
Fluoxetine	1 (10)	0 (0)	NA
Diltiazen	1 (10)	0 (0)	NA
Enalapril	3 (30)	5 (55)	NA
Losartan	1 (10)	1 (11)	NA
Anlodipine	0 (0)	2 (22)	NA
Carbamazepine	1 (10)	0 (0)	NA
Methylphenidate	0 (0)	0 (0)	NA
Clopidogrel	0 (0)	0(0)	NA
Warfarin	1(10)	0(0)	NA
Acetylsalicylic Acid	1 (10)	0 (0)	NA

Results are presented as mean (SD) unless stated otherwise. C-SLE, childhood-onset systemic lupus erythematosus; SLEDAI, systemic lupus erythematosus disease activity index; SLICC/ACR-DI, Systemic Lupus International Collaborating Clinics/ACR Damage Index; NA, not applicable.

### Exercise tolerance and cardiorespiratory measurements at baseline

The exercise and cardiorespiratory parameters at baseline are shown in Table 2. The C-SLE patients presented lower time-to-exhaustion, peak speed, peak VO_2 _and CR during the cardiorespiratory exercise test and higher resting HR when compared with the healthy control subjects (*P *< 0.05, between-group comparisons). In addition, the relative change in HR (Δ%) from rest to VAT, rest to RCP, and rest to peak of exercise, and the HR recovery (that is, ΔHRR1 and ΔHRR2) were also significantly lower in the C-SLE patients when compared with healthy controls (*P *< 0.05, between-group comparisons). Importantly, all of the parameters were similar between the TR and NT groups (*P *> 0.05, between-group comparisons).

**Table 2:** Exercise tolerance and cardiorespiratory measurements before and after the exercise training

Variable	C-SLE trained (*n *= 10)	C-SLE non-trained (*n *= 9)	Control (*n *= 10)
Time-to-exhaustion, minutes			
PRE	10.1 (2.2)^a^	9.7 (1.6)^a^	13.7 (2.7)^b^
POST	12.8 (2.4)^b^	10.1 (1.8)^a^	---
Resting heart rate, bpm			
PRE	102.6 (10.3)^a^	106.1 (12.1)^a^	90.5 (15.7)^b^
POST	86.2 (13.8)^b^	110.6 (8.4)^a^	---
Peak heart rate, bpm			
PRE	178.2 (18.3)^a^	177.6 (17.4)^a^	195.8 (8.9)^a^
POST	183.6 (10.5)^a^	178.1 (18.6)^a^	---
Peak VO_2_, ml/Kg/min			
PRE	27.7 (6.1)^a^	25.7 (3.1)^a^	39.9 (7.3)^b^
POST	31.8 (8.0)^b^	25.8 (4.2)^a^	---
Peak speed (Km/h)			
PRE	4.4 (0.6)^a^	4.2 (0.5)^a^	5.4 (0.9)^b^
POST	5.2 (0.8)^b^	4.3 (0.5)^a^	---
Chronotropic reserve, %			
PRE	75.5 (18.7)^a^	74.8 (17.3)^a^	96.6 (8.2)^b^
POST	87.1 (9.0)^b^*	74.1 (18.9)^a^	---
Heart rate, Δ%			
Rest to VAT			
PRE	29.8 (16.6)^a^	25.3 (12.0)^a^	60.0 (24.8)^b^
POST	56.3 (18.7)^b^	17.0 (9.5)^a^	---
Rest to RCP			
PRE	53.9 (13.7)^a^	54.9 (24.9)^a^	97.3 (35.9)^b^
POST	97.4 (28.6)^b^	42.8 (17.4)^a^	---
Rest to Peak			
PRE	75.3 (24.8)^a^	68.9 (22.9)^a^	122.6 (42.1)^b^
POST	117.7 (34.3)^b^	61.5 (16.6)^a^	---
Heart rate recovery, ΔHRR			
ΔHRR1			
PRE	25.5 (14.9)^a^	26.6 (10.0)^a^	40.2 (6.9)^b^
POST	41.5 (10.0)^b^	26.3 (8.0)^a^	---
ΔHRR2			
PRE	36.6 (10.2)^a^	35.6 (11.8)^a^	57.8 (8.9)^b^
POST	62.2 (13.7)^b^	39.8 (9.7)^a^	---

Data are expressed as means (SD) (mixed model for repeated measures). Relative change (Δ%) for heart rate was calculated for the intervals between rest to ventilator anaerobic threshold (VAT), rest to respiratory compensation point (RCP), and rest to peak of exercise. Absolute change (Δ) was used to calculate the difference between heart rate at peak exercise and at the first (ΔHRR1) and second minutes (ΔHRR2) after the exercise test. Means with different letters are significantly different from each other (*P *< 0.05, between- or within-group comparisons). *P = 0.06, within-group comparison. C-SLE, childhood-onset systemic lupus erythematosus; PRE, pre-intervention (baseline), POST, post-intervention; Peak, peak exercise; VO_2_, oxygen consumption.

### Exercise tolerance and cardiorespiratory measurements after the exercise training program

**Figure 2:**
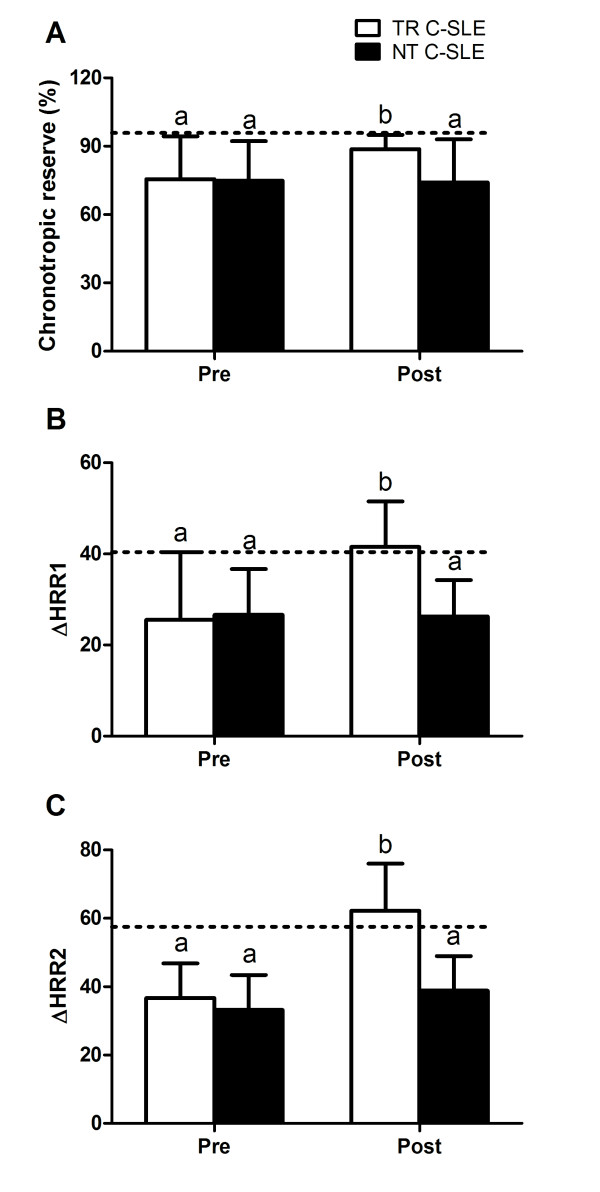
**Cardiorespiratory measurements before (PRE) and after (POST) the exercise training**. Dotted lines represent mean data from the healthy control group at baseline. (**A**) Chronotropic reserve. (**B**) Absolute change in heart rate at the first minute after exercise (ΔHRR1). (**C**) Absolute change in heart rate at the second minute after exercise (ΔHRR2). TR C-SLE, trained patients with childhood-onset systemic lupus erythematosus; NT C-SLE, non-trained patients with childhood-onset systemic lupus erythematosus. Means with different letters are significantly different from each other: a, significant difference when compared with the healthy control group; b, no significant difference when compared with the healthy control group.

**Figure 3:**
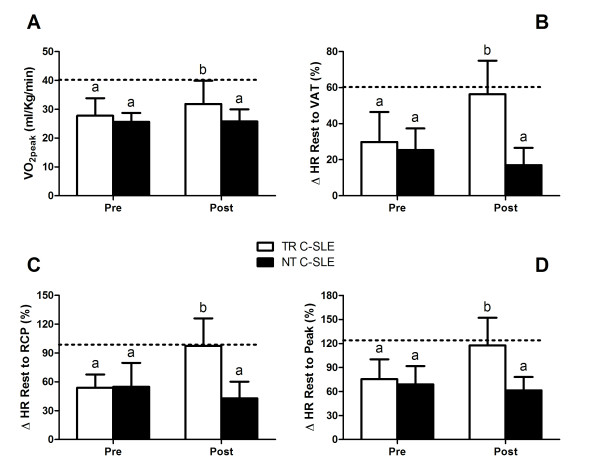
**Cardiorespiratory measurements before (PRE) and after (POST) the exercise training**. Dotted lines represent mean data from the healthy control group at baseline. (A) Peak oxygen consumption (VO_2 _peak). (**B**) Relative change in heart rate (Δ%) between rest and the ventilatory anaerobic threshold (VAT). (**C**) Relative change in heart rate (Δ%) between rest and the respiratory compensation point (RCP). (**D**) Relative change in heart rate (Δ%) between rest and peak exercise (Peak). TR C-SLE, trained patients with childhood-onset systemic lupus erythematosus; NT C-SLE, non-trained patients with childhood-onset systemic lupus erythematosus. Means with different letters are significantly different from each other: a, significant difference when compared with the healthy control group; b, no significant difference when compared with the healthy control group.

The exercise and cardiorespiratory parameters after the exercise training program are shown in Table 2. As expected, the NT group did not present any change in any of the parameters (*P *< 0.05, within-group comparisons). In contrast, the TR group presented significant increases in time-to-exhaustion (*P *= 0.01, ES = 1.07) and peak speed (*P *= 0.01, ES = 1.08) and a decrease in resting HR (*P *= 0.0002, ES = -1.45) when compared with the NT group. Additionally, the TR group had increased peak VO_2 _(*P *= 0.04, ES = 0.86), CR (*P *= 0.06, ES = 0.83), relative change in HR (Δ%) from rest to VAT (*P *= 0.0001, ES = 1.58, rest to RCP (*P *= 0.0001, ES = 1.49), and rest to peak of exercise (*P *= 0.0003, ES = 1.53), and ΔHRR1 (*P *= 0.003, ES = 1.29) and ΔHRR2 (*P *= 0.0008, ES = 1.36) in comparison to the NT group. Moreover, after the aerobic exercise training program, the latter cardiorespiratory responses to exercise were comparable between the trained group and the healthy control group (*P *> 0.05) (Figures 2 and 3).

### Clinical parameters, drug regimen, and adverse effects

SLEDAI-2K scores remained unchanged throughout the study for both groups (TR: PRE = 5.0 ± 5.1, POST = 3.8 ± 3.8, *P *= 0.71, and NT: PRE = 5.5 ± 6.4, POST = 3.1 ± 3.8, *P *= 0.20; within-group comparison).

Prednisone dosage was also unchanged in both groups after the intervention (TR: PRE = 14.8 ± 11.1, POST = 12.5 ± 7.2 mg/day, *P *= 0.39, and NT: PRE = 13.8 ± 6.5, POST = 12.5 ± 5.8 mg/day, *P *= 0.84; within-group comparisons). Similarly, the other drugs (type and dose of medications) remained stable throughout the study. Additionally, the levels of ESR, CRP, C3 and C4 and anti-DNAds antibodies were unaltered in both groups throughout the study (*P *> 0.05, within-group comparison; data not shown). Finally, there was no clinical evidence of excessive exhaustion, pain, osteoarticular injury, muscle soreness, or any other adverse event.

### BMD and body composition

The TR, NT and C groups were similar in body composition and BMD parameters at baseline (*P *> 0.05, between-group comparisons). Additionally, no significant changes were observed in any of these parameters after the intervention (*P *> 0.05, within-group comparisons) (Table 3).

**Table 3:** Body composition, bone mineral apparent density (BMAD) and bone mineral content (BMC) before and after exercise training

Variable	C-SLE trained (*n *= 10)	C-SLE non-trained (*n *= 9)	Control (*n *= 10)
BMAD, L1-L4, g/cm^3^			
PRE	0.097 (0.013)	0.108 (0.009)	0.113 (0.016)
POST	0.098 (0.012)	0.108 (0.010)	---
BMAD, TF, g/cm^3^			
PRE	0.133 (0.019)	0.145 (0.017)	0.163 (0.032)
POST	0.134 (0.020)	0.145 (0.021)	NA
BMAD, WB without head, g/cm^3^			
PRE	0.023 (0.001)	0.025 (0.002)	0.025 (0.002)
POST	0.024 (0.002)	0.024 (0.002)	NA
Total BMC, g			
PRE	1,223.0 (270.4)	1,186.2 (271.2)	1,530.0 (567.7)
POST	1.245.0 (262.7)	1,221.2 (252.7)	NA
BMC, without head, g			
PRE	910.0 (233.1)	873.8 (267.4)	1,168.3 (508.0)
POST	927.0 (231.1)	916.3 (242.1)	NA
Lean mass, Kg			
PRE	31.5 (4.9)	29.9 (7.6)	33.9 (10.8)
POST	31.8 (5.1)	30.7 (7.4)	NA
Fat mass, Kg			
PRE	17.4 (7.3)	16.8 (6.0)	11.7 (7.2)
POST	17.0 (7.9)	19.1 (6.3)	NA
Height, m			
PRE	1.48 (0.09)	1.43 (0.9)	1.51 (0.13)
POST	1.49 (0.09)	1.44 (0.1)	NA
Weight, Kg			
PRE	48.7 (10.1)	49.7 (12.1)	46.6 (14.5)
POST	49.7 (11.3)	51.8 (13.3)	NA

Data are expressed as means (SD) (mixed model for repeated measures). C-SLE, childhood-onset systemic lupus erythematosus; PRE, pre-intervention (baseline), POST, post-intervention; BMAD, bone mineral apparent density; BMC, bone mineral content; TF, total femur; L1-L4, lumbar vertebrae L1 to L4; WB, whole body; NA, not applicable. There were no significant differences between or within groups for any of the variables.

## Discussion

To the best of our knowledge, this is the first randomized controlled trial study aimed at investigating the effects of an exercise training program in a cohort of C-SLE patients. The main finding of this study is that a supervised aerobic exercise training program was safe and effective in improving the cardiorespiratory capacity in C-SLE patients.

C-SLE is commonly associated with poor physical function and low levels of physical activity [11,12], possibly as a consequence of reduced cardiorespiratory capacity [8-12] and chronic fatigue [12]. Supporting this concept, we observed lower cardiorespiratory capacity (for example, lower peak VO_2_) and exercise tolerance (for example, lower time-to-exhaustion in the cardiorespiratory exercise test) in C-SLE patients when compared with their healthy peers. Moreover, we showed for the first time that C-SLE patients presented an abnormal HR response during and after the cardiorespiratory test (for example, low CR and delayed HR recovery), which are considered as important non-invasive markers of autonomic dysfunction [37-39]. Our results are in accordance with previous studies conducted with adult SLE patients where the same abnormal pattern was evidenced [9,39]. Notably, there is evidence showing that, at least in adult cohorts, low cardiorespiratory fitness and dysautonomia are associated with all-cause mortality and increased cardiovascular risk [40,41]. However, these relationships remain to be proven in SLE cohorts. In this context, strategies capable of improving the cardiorespiratory capacity and counteracting autonomic dysfunction are of upmost importance in the management of C-SLE patients who are already at increased CVD risk [3,4].

Exercise has been shown to be a potential therapeutic tool in the management of adult SLE. Three pilot studies demonstrated that supervised exercise training can alleviate fatigue in adult SLE patients [8,14,15]. Furthermore, Tench *et al. *[42] observed improvements in fatigue after a three-month home-based exercise program. Similarly, Carvalho *et al. *[16], reported beneficial effects of a three-month exercise program on exercise tolerance, aerobic conditioning (for example, increased VO_2max_), and quality of life in SLE patients aged 18 to 55 years. Although these studies point out the potential therapeutic role of exercise in SLE, some limitations (such as lack of a randomized control design and small sample sizes) preclude definitive conclusions. Moreover, studies aimed at investigating the effects of exercise on physical capacity in pediatric rheumatologic patients are still scarce. Nonetheless, we have recently shown that a three-month combined endurance and resistance exercise training program induced improvements in the cardiorespiratory capacity (for example, increased VO_2 _peak and time-to-exhaustion), muscle strength, and physical function in patients with JDM [23]. Furthermore, we also demonstrated that a three-month supervised aerobic exercise training program was capable of increasing VO_2 _peak and time-to-exhaustion in a 15-year-old boy with antiphospholipid syndrome and C-SLE [25]. Importantly, the current findings extend the previous observations on the benefits of exercise in adult SLE and other pediatric rheumatologic patients to C-SLE.

Specifically, we showed that a three-month aerobic training program was effective in improving resting HR and peak VO_2_, peak speed, and time-to-exhaustion in a maximal graded exercise test in C-SLE patients. Noticeably, after the exercise training program, these parameters were comparable with those of their healthy peers, suggesting that the exercise training was effective in offsetting the C-SLE-related impairment in physical function and aerobic capacity.

Accordingly, we demonstrated that the exercise training program was also effective in attenuating the impaired CR and the delayed post-exercise HR recovery in C-SLE patients, supporting the homeostatic role of exercise in C-SLE, particularly regarding the autonomic dysfunction. The present results corroborate the positive effects of exercise training upon autonomic control in adult patients with SLE [9] and CVD [43].

Children display a high bone turnover rate and a more pronounced change in bone mass in response to exercise training than later in life. In fact, we have previously demonstrated that a short-term (12 weeks) exercise training program comprising aerobic and resistance exercises was sufficient to increase BMAD in JDM patients [23]. Conversely, no changes in BMAD were observed in the present study. This result is somewhat expected as the C-SLE patients underwent only moderate-intensity aerobic exercise, which is unlikely to provide sufficient stimulus to promote bone mass accretion. Therefore, future longer-term studies involving a more intensive aerobic and resistance training exercises should be performed to comprehensively examine the role of exercise on bone mass in C-SLE patients.

Finally, it is important to emphasize that the exercise training program did not provoke disease flare, as evidenced by lack of changes in disease activity scores and laboratory parameters of inflammation. Additionally, the drug regime was virtually unchanged throughout the study and no other adverse events were reported. Collectively, these findings suggest that a supervised moderate-intensity aerobic training program may be safe for C-SLE patients, supporting previous studies with adult SLE patients [8,9,14,15].

This study presents some limitations. First, our sample may be considered heterogeneous since the patients presented a large range of active disease scores. The selection of patients with different active disease scores was intentional and aimed at increasing the external validity of our study. However, we acknowledge that the number of patients enrolled in this trial did not allow us to compare the potential differential responses of active and non-active subjects to the exercise training. Therefore, further studies with larger samples comparing the exercise responses as a function of active disease are indeed necessary. Second, the healthy children did not participate in the exercise training program, thus precluding any comparison between patients and controls with respect to adaptive responses to exercise (for example, magnitude of changes). Finally, this was a short-term intra-hospital supervised intervention. Further studies should investigate the efficacy, safety and feasibility of other training program (such as, recreational physical activity, sports-related activities, physical education classes, and home-based programs). Moreover, it is imperative to investigate the long-term effects of exercise (or spontaneous physical activity) in more robust outcomes (for example, incidence of institutionalization, disease flares, health-related quality of life, cardiovascular events and lipid and bone metabolism).

## Conclusions

In conclusion, this study demonstrated for the first time that a three-month supervised moderate-intensity aerobic exercise training program can be safe and effective in ameliorating the cardiorespiratory capacity and the autonomic function in C-SLE patients. These findings stress the potential role of exercise training in the management of C-SLE, strengthening previous evidence of the beneficial effects of exercise in other pediatric rheumatic diseases.

## Abbreviations

BA: bone area; BMAD: bone mineral apparent density; BMC: bone mineral content; BMD: bone mineral density; BMI: body mass index; C: healthy control group; C3: complement 3; C4: complement 4; CR: chronotropic reserve; CRP: C-reactive protein; C-SLE: childhood-onset systemic lupus erythematosus; CVD: cardiovascular disease; ΔHRR: heart rate recovery; ΔHRR1: heart rate recovery at the first minute; ΔHRR2: heart rate recovery at the second minute; DXA: dual-energy x-ray absorptiometry; ES: effect size; ESR: erythrocyte sedimentation rate; HR: heart rate; JDM: juvenile dermatomyositis; NT: non-trained C-SLE group; POST: 12 weeks post-intervention; PRE: pre-intervention (baseline); RCP: respiratory compensation point; RER: respiratory exchange ratio; SLE: systemic lupus erythematosus; SLEDAI: systemic lupus erythematosus disease activity index; SLICC/ACR-DI: Systemic Lupus International Collaborating Clinics/ACR Damage Index; TR: trained C-SLE group; VAT: ventilatory anaerobic threshold; VO_2_: oxygen consumption; VO_2 _peak: peak oxygen consumption.

## Competing interests

The authors declare that they have no conflicts of interest.

## Authors' contributions

Study conception and design: DMLP, CAS, BG, ALSP, EB, and HR. Acquisition of data: DMLP, CAS, AMES, RMRP, APH, and FBB. Analysis and interpretation of data: DMLP, CAS, AEMS, FBB, RMRP, ALSP, APH, BG, EB, and HR. All of the authors were involved in drafting the article or revising it critically for important intellectual content, and all of the authors approved the final version to be submitted for publication. The authors had full access to all of the data in the study and take responsibility for the integrity of the data and the accuracy of the data analysis.
